# Calcium Dyshomeostasis in Alzheimer’s Disease Pathogenesis

**DOI:** 10.3390/ijms22094914

**Published:** 2021-05-06

**Authors:** Roberta Cascella, Cristina Cecchi

**Affiliations:** Department of Experimental and Clinical Biomedical Sciences, University of Florence, 50134 Florence, Italy; roberta.cascella@unifi.it

**Keywords:** protein aggregation, amyloid β peptide (Aβ), toxic oligomers, amyloid fibrils, tau protein, neurodegeneration, ionic dysregulation, glutamatergic receptors, NMDA, AMPA

## Abstract

Alzheimer’s disease (AD) is the most common age-related neurodegenerative disorder that is characterized by amyloid β-protein deposition in senile plaques, neurofibrillary tangles consisting of abnormally phosphorylated tau protein, and neuronal loss leading to cognitive decline and dementia. Despite extensive research, the exact mechanisms underlying AD remain unknown and effective treatment is not available. Many hypotheses have been proposed to explain AD pathophysiology; however, there is general consensus that the abnormal aggregation of the amyloid β peptide (Aβ) is the initial event triggering a pathogenic cascade of degenerating events in cholinergic neurons. The dysregulation of calcium homeostasis has been studied considerably to clarify the mechanisms of neurodegeneration induced by Aβ. Intracellular calcium acts as a second messenger and plays a key role in the regulation of neuronal functions, such as neural growth and differentiation, action potential, and synaptic plasticity. The calcium hypothesis of AD posits that activation of the amyloidogenic pathway affects neuronal Ca^2+^ homeostasis and the mechanisms responsible for learning and memory. Aβ can disrupt Ca^2+^ signaling through several mechanisms, by increasing the influx of Ca^2+^ from the extracellular space and by activating its release from intracellular stores. Here, we review the different molecular mechanisms and receptors involved in calcium dysregulation in AD and possible therapeutic strategies for improving the treatment.

## 1. Alzheimer’s Disease

Alzheimer’s disease (AD) is the most common cause of age-related neurodegenerative disease, which is characterized by progressive memory loss, cognitive dysfunction, language disorders, and personality changes [1]. While there is no cure or a way to stop or slow AD progression, there are drug and non-drug options that may help to treat symptoms [2,3]. In particular, the FDA-approved drugs are acetylcholine esterase (AChE) inhibitors and N-methyl D-aspartate receptor (NMDAR) blocker, but they cause a variety of side effects [4,5,6]. In 2015, around 46.8 million individuals worldwide had dementia, increasing to 50 million people in 2017 and expecting to rise exponentially in the next few years [7]. Numerous research studies have tried to elucidate the mechanisms of the pathogenesis and development of this disease, and multiple hypotheses have been postulated. The pathogenesis of AD involves the massive extracellular deposition of amyloid-β (Aβ), forming cores of senile plaques in the brain parenchyma, and intracellular accumulation of the abnormally hyperphosphorylated tau proteins, forming neurofibrillary tangles (NFTs) [2,8,9,10,11]. Aβ and NFTs induce the loss of neurons and synaptic density by enhancing the inflammation process, oxidative stress and the occurrence of cerebral microvascular disease [12]. Aβ plaque also promotes the senescence of neural stem/progenitor cells by affecting forebrain and hippocampal neurogenesis [13]. Emerging evidence suggests the existence of additional molecular pathophysiological pathways, including axonal disintegration [14], synaptic dysfunction and degeneration [15], innate immune responses and neuroinflammation [16,17], vascular dysregulation [12,18], and brain metabolic dysfunction [19] across the different stages of AD.

Although extracellular Aβ plaques and NFTs in the brain are hallmarks of AD, the multiple mechanisms related to the disease are still unclear. The amyloid cascade was considered for a long time as a dominant model for AD pathogenesis [10]. According to this hypothesis, the starting event in AD is the aggregation and subsequent deposition of the Aβ peptide in the brain [20,21], resulting in the hyperphosphorylation of tau into NFTs and, ultimately, the degeneration of neurons. This hypothesis is supported by the identification of mutations in the genes encoding amyloid precursor protein (APP) and the presenilin proteins (PS1 and PS2), causing an overproduction of Aβ or an increase in its aggregation potential [22,23,24]. More recently, accumulating evidence suggests that the hyperphosphorylation and polymerization of tau into NFTs have a synergistic effect with Aβ on AD pathogenesis [25].

There are two major forms of AD: the sporadic or late-onset form (SAD), the most common one, and the familial or early-onset form (FAD), representing less than 5% of the cases [26]. Although there are genetic and pathological differences between the two forms of AD, they show remarkable similarities in their pathophysiology and clinical symptoms [27]. Studies on animal models carrying APP, PS1 or PS2 mutations suggest that intervention at the embryonic stage is beneficial for inducing synaptic plasticity [28]. Nevertheless, while APP, PS1 and PS2 gene mutations were identified as responsible for autosomal-dominant AD, the etiology of SAD still remains elusive. Indeed, SAD appears to be influenced by the combined action of multiple genetic susceptibilities and environmental risk factors [29]. In addition, early diagnosis or intervention aimed at the high-risk factors, such as type 2 diabetes mellitus and hyperhomocysteinemia, may be suitable [30,31,32,33]. One of the major genetic risk factors of SAD is the Epsilon 4 (ε4) allele of the apolipoprotein E gene (APOE4), which is involved in the aggregation and clearance of Aβ and in cholesterol homeostasis [34]. Different polymorphic forms of APOE4 have been reported and, among them, the ε4 form was found to correlate with a major risk of AD, both in homozygosis and heterozygosis [35]. Structural and functional neuroimaging studies found hippocampal and medial-temporal lobe atrophies in both types of AD, which also share temporoparietal hypometabolism and sporadic memory and judgment impairment. In addition, myoclonus and seizures are also frequently observed. Regarding symptoms, late-onset forms show typical manifestations of dementia, with memory impairment and executive dysfunction interfering with daily life activities. On the other hand, early-onset forms have atypical symptoms, including language, visual, practice, or executive problems, that appear more pronounced with respect to memory deficits [9].

### Aβ Peptide and Tau Protein: Synergistic Effects

The Aβ peptide is generated from the endosomal proteolytic cleavage of the APP by γ-secretase and β-site APP cleaving enzyme 1 (BACE1), which catalyzes the rate-limiting step [36]. During the intracellular trafficking of APP into the endocytic compartment from the plasma membrane, APP is first cleaved by BACE1, followed by γ-secretase cleavage of the stub of APP at different positions, leading to the production of C-terminal ends of Aβ of various lengths such as Aβ40 and Aβ42 [37,38]. Aβ42, with respect to Aβ40, is more hydrophobic, prone to amyloid formation, and is the initial and predominant species found in senile plaques [39]. In healthy brains, the non-amyloid pathway requires APP to be first cleaved by α-secretase, producing an extracellular secretory fragment (sAPPa) and a membrane-bound carboxy-terminal fragment, which is then cleaved by γ-secretase into small fragments that can be completely degraded. Jonsson and coworkers showed that the A672T mutation in the APP gene causes a decrease in the β-cleavage of APP, resulting in protection against AD [40]. By contrast, several gene mutations on APP have been identified to promote Aβ production. In addition, various genetic studies have demonstrated that autosomal-dominant familial AD mutations in PS1 and PS2, the catalytic subunit of γ-secretase, cause the increase in Aβ42 production, suggesting the involvement of γ-secretase in the pathogenesis of AD [41,42,43,44]. During endocytosis from the plasma membrane, γ-secretase cleaves its substrate C99, which is the C-terminal fragment of APP, to generate Aβ in the endosomes. Recently, it has been reported that phosphatidylinositol binding clathrin assembly protein (PICALM), a genetic risk factor of AD, affects Aβ production through the regulation of the subcellular localization of γ-secretase [45]. Following the production of Aβ peptides through the cleavage of APP by β and γ secretases and the secretion in the external environment, an aggregation process produces a variety of oligomeric forms that act as active neurotoxins, causing neuronal dysfunction, the loss of synaptic connections, and cell death. In particular, Aβ peptides undergo a conformational change, forming β-sheet-rich oligomeric complexes of different sizes that eventually develop into amyloid-type fibrils forming cores of senile plaques in the brain parenchyma [46,47,48].

The tau protein is an axonal microtubule-associated protein that is distributed in the axons of neurons and is one of the main components of the cytoskeleton [49]. Tau is known to undergo several post-translational modifications, such as phosphorylation, acetylation, methylation, glycation, polyamination, glycosylation, nitration, ubiquitination, sumoylation, isomerization, and oxidation, most of which occur at multiple residues along the protein [50]. The most studied post-translational modification of tau is phosphorylation, as its abnormal phosphorylation is associated with several tauopathies [49]. The hyperphosphorylation of tau is known to be involved in the onset and progression of AD, leading to the aggregation and development of NFTs commonly found inside neurons of AD patients [51]. The formation of cytoplasmic NFTs compromises microtubules and causes the disruption of several cellular pathways, including proliferation, differentiation and protein trafficking [52]. Numerous research data from multiple laboratories demonstrated that misfolded tau can be released from neurons and taken up by connected cells, leading to a spreading process between cells that might then recruit endogenous tau to the misfolded state [53]. This model is in agreement with those of α-synuclein in Parkinson’s disease and other neurodegenerative disorders [53,54].

A dominant theory for the incidence of AD is the “amyloid hypothesis”, which describes a complex sequence of pathogenic events responsible for neurodegeneration [55,56,57,58,59,60]. According to this hypothesis, the aberrant accumulation of the Aβ peptide, following the amyloidogenic processing of the APP, results in the production of cytotoxic complexes and its deposition in various brain areas. This accumulation triggers a cascade of pathogenic events, including the alteration of ionic homeostasis, oxidative stress, inflammation and vascular damage. Aβ complexes are also responsible for a wide range of biochemical and structural changes in the nearby neurites and cell bodies, culminating in neuronal dysfunction and synapse loss [61]. AD pathogenesis has been prevalently associated with the progressive Aβ accumulation in the brain parenchyma and the formation of senile plaques. The presence of Aβ peptides in senile plaques of AD patients [62], as well as the location of the APP gene on chromosome 21, which causes Down’s syndrome [20], have originally reinforced the amyloid cascade hypothesis. Nevertheless, recent evidence suggests that the Aβ peptide can act as a “seed,” in the development rather than the progression of the disease [63]. In addition, the amyloid hypothesis was extensively criticized because of many reasons, including that APP mutations in AD patients have a low frequency, many Down’s syndrome patients did not develop AD and the presence of presenilin mutations did not correlate with the increased Aβ production [64]. Furthermore, the Aβ peptide is an important metabolite whose beneficial function should be identified together with that of APP, and Aβ deposition does not correlate with the severity of dementia as along with tangle density or synapse loss. Further investigations in AD mouse models demonstrated that the accumulation of Aβ fibrils in senile plaques did not correlate with neuronal cell death [65], suggesting that Aβ oligomers might be the key cytotoxic agents rather than the fibrillar form. Indeed, even though the Aβ peptide has been first identified as the main component of the extracellular amyloid plaques, it is now well established that the oligomeric species accumulating intracellularly, as dimers and trimers, are more toxic than the extracellular Aβ fibrils [66]. Moreover, several studies demonstrated that Aβ oligomers have a key role in synaptic dysfunction and neuronal alterations [67,68,69,70], leading to cognitive impairment many years before the formation of amyloid plaques and neuronal death [71,72]. The specific mechanisms for Aβ peptide-induced cytotoxicity have not yet been completely elucidated. Several studies have suggested that the reuptake of extracellular Aβ into neurons may lead to neuronal damage and neurotoxicity [73]. The toxicity of Aβ oligomers involves the alteration of the plasma membrane [74,75,76], ion dysregulation [74,77,78], oxidative stress [79,80,81,82,83,84,85], the inhibition of proteasomal degradation [86,87], the impairment of autophagy [87,88] and inflammation [89,90,91]. However, the multifactorial nature of AD points out that the Aβ peptide might be required but not sufficient for the development of the disease [92]. This could explain the failure of anti-Aβ clinical trials and suggest that the role of tau needs to be reconsidered [25].

According to the “tau hypothesis”, the intracellular NFTs, mainly composed of abnormally phosphorylated and aggregated tau proteins, are considered as the principal effectors of neuronal loss and memory impairment in AD via the impairment of axonal transport. In contrast to the view that there is no particular interaction between Aβ and tau, recent experimental and clinical evidence supports a strong Aβ–tau synergy [25]. Indeed, the presence of Aβ was found to enhance tau phenotypes throughout the disease course [93], and the functional consequences of such interplay occur in late stages of the disease [94]. In particular, the researchers noticed that the propagation of tau is always associated with the presence of Aβ plaques [54]. Furthermore, in human postmortem tissues, the presence of Aβ was found to promote the formation of a specific form of hyperphosphorylated tau, which is particularly prone to spread [95]. These data are in agreement with PET results showing that tau accumulation in the cortex of cognitively normal older individuals was accelerated in the presence of Aβ [96]. The synergistic association between Aβ and tau was also demonstrated in the CSF, where both total tau and phosphorylated tau levels correlated with cognitive performance only when Aβ deposition was contemporaneously present [97]. Thus, neither the amyloid nor tau hypotheses are sufficient to explain all the pathological mechanisms responsible for AD pathogenesis [25,98], but rather a unique theory taking into account the synergistic effects of both would explain many pathogenic processes occurring during AD progression.

## 2. Regulation of Ionic Homeostasis

The regulation of ionic homeostasis is crucial for several neuronal functions. Indeed, ion gradients provide the driving force for important intra- and inter-cellular communications within neuronal networks. In particular, sodium (Na^+^) entry into neurons is essential for the propagation of action potentials, whereas Ca^2+^ signaling is involved in neurotransmitter release, synaptic plasticity, gene expression and other important neuronal functions [99,100,101]. Ca^2+^ concentration is finely regulated by cell surface receptors, channels, pumps, antiporters, Ca^2+^ buffers, and Ca^2+^ sensors. These components have specific distributions and roles within the cell, contributing to the maintenance of intracellular Ca^2+^ homeostasis [102]. The efflux of potassium (K^+^) ions through specific channels, instead, mediates the repolarization of membrane potential following depolarization. Increasing evidence indicates that the progressive Aβ overproduction and accumulation cause the dysregulation of ionic homeostasis. Indeed, it has been widely demonstrated that Aβ accumulation causes the influx of Ca^2+^ from the extracellular space, leading to Ca^2+^ dyshomeostasis [75,103]. On the contrary, intracellular Ca^2+^ levels can modulate APP processing and Aβ production as well as the formation of NFTs [104,105]. Importantly, the genes associated with the development of AD have also been found to modulate Ca^2+^ signaling. Nonetheless, many K^+^ and Na^+^ channels appeared downregulated in both AD patients and experimental models, suggesting their potential role in AD pathophysiology. Moreover, the activity of ion-motive ATPases was found to be impaired in AD brains [106,107], thus contributing to the dyshomeostasis of Na^+^ and Ca^2+^ by inducing membrane depolarization and the opening of voltage-sensitive channels. All these disruptions contribute to the activation of intracellular pathways, leading to neuronal dysfunction and death [108].

### Calcium Homeostasis

Calcium ions (Ca^2+^) regulate the function of various enzymes and proteins and play an important role as secondary messengers in signal transduction pathways, including cell survival, proliferation, differentiation and apoptosis [109]. Ca^2+^ is also involved in the regulation of multiple neuronal and astrocytic functions, such as neurotransmitter release, synaptic plasticity, membrane excitability, gene transcription, proliferation and cell death [110,111]. It binds calmodulin (CaM), causing its conformation change and the activation of calcineurin (CaN), Ca^2+^/calmodulin dependent protein kinase II (CaMKII) and IV (CamKIV). CaMKII plays a pivotal role in synaptic strengthening [112], whereas CaMKIV regulates the transcription of cAMP response element binding protein (CREB), which is implicated in memory formation [113]. Ca^2+^ also modulates the function of protein kinase C (PKC), thus regulating cell survival and cell division [109].

**Figure 1:**
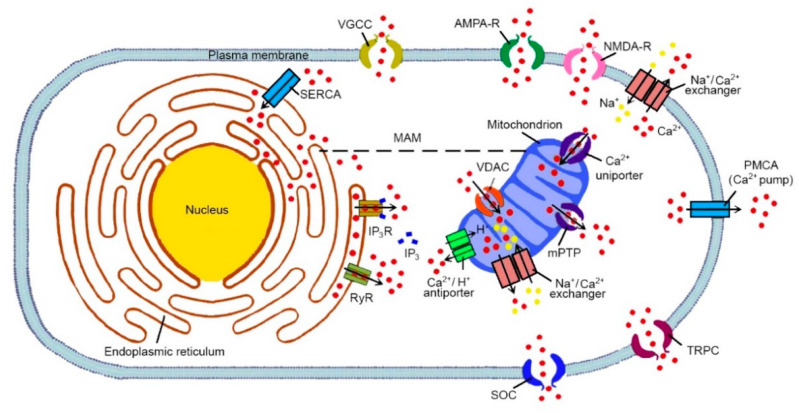
Calcium homeostasis in normal cells. Intracellular calcium (Ca^2+^) levels are finely regulated within their physiological range (10–100 nM) against steep gradients by transport of the ion to the extracellular space and cisternae of the ER, and by protein binding. Cellular calcium influx through the plasma membrane is largely mediated by different types of Ca^2+^ channels (NMDA-R, AMPA-R, VGCC, SOC and TRPC channels) and, under exceptional circumstances, including strong depolarization or the presence of high intracellular sodium (Na^+^) concentrations, the Na^+^/Ca^2+^ exchanger. Ca^2+^ may also be released into the cytoplasm from the ER, through IP_3_R and RYR. Cytosolic Ca^2+^ increase is counterbalanced by different systems. In particular, the PMCA, Na^+^/Ca^2+^ exchangers, and SERCA restore physiological calcium levels. The excess of intracellular Ca^2+^ can also be taken up by mitochondria through mitochondrial Ca^2+^ uniporters and VDAC. Ca^2+^ can be also released back into the cytosol through the mitochondrial Na^+^/Ca^2+^ exchangers, which can also reverse its mode of operation, allowing the Ca^2+^ entry into the mitochondrial matrix, the Ca^2+^/H^+^ antiporter and mPTP.

The concentration of cytoplasmic Ca^2+^ in resting condition is maintained at ~100 nM, far below the endoplasmic reticulum (ER) (100–800 μM) and the extracellular medium (~1–2 mM) [114], and can increase up to 1–3 µM upon cell stimulation. Indeed, Ca^2+^ can cross the cellular membrane and/or can be released from intracellular stores [109]. These levels are finely regulated in cellular compartments by a different array of receptors, calcium channels and calcium pumps (Figure 1).

A variety of Ca^2+^ channels are present in the plasma membrane with a different distribution, resulting in a fine regulation of its concentration between the intracellular and extracellular space. In particular, N-methyl-D-aspartate receptor (NMDA-R), α-amino-3-hydroxy-5-methyl-4-isoxazolepropionic acid receptor (AMPA-R), voltage-gated calcium channel (VGCC) and transient receptor potential cation channels (TRPC) regulate the Ca^2+^ influx from the extracellular space through a variety of signaling mechanisms [115]. The other major source for intracellular Ca^2+^ comes from internal stores, mostly the ER. The flux of Ca^2+^ from the ER to the cytosol is regulated by two key intracellular Ca^2+^ releasing channels: the inositol 1, 4, 5-trisphosphate receptor (IP_3_R) [116] and the ryanodine receptor (RyR) [117].

The first mechanism is driven by the G-protein coupled receptors (GPCRs) that activate phospholipase C (PLC), thus mobilizing the secondary messenger IP_3_ and diacylglycerol (DAG). IP_3_ interacts with IP_3_R on ER, causing its opening and the release of Ca^2+^ from the lumen of the ER to the cytosol. Released cytosolic Ca^2+^ regulates, in a specific concentration-dependent manner, the IP_3_R opening and the activation of RyRs, causing a further release of Ca^2+^ from the ER (Figure 1) [118]. In addition, the Ca^2+^ depletion in the ER causes the activation of the store operated channel (SOC) pathway, which sequesters Ca^2+^ from the extracellular space [119]. Further, sarco-endoplasmic reticulum calcium transport ATPase (SERCA) pumps, which are located on the ER surface, take up the Ca^2+^ from the cytoplasm to the ER [120]. Resting Ca^2+^ concentrations were maintained via extrusion mechanisms that transport Ca^2+^ out of the cell or back into intracellular stores. Indeed, Ca^2+^ is extruded from the cell by the plasma membrane Ca^2+^ATPase (PMCA) [121] and the Na^+^/Ca^2+^ exchanger (NCX) [122]. Mitochondria also play a key role in maintaining cytosolic Ca^2+^ homeostasis (Figure 1). Indeed, mitochondria can activate both Ca^2+^ uptake and release through the mitochondrial Ca^2+^ uniporter complex (MCUC) [123,124], the Ca^2+^/Na^+^ antiporter (NCLX) [125], the Ca^2+^/H^+^ antiporter [126], the voltage-dependent anion selective channel protein (VDAC) and the mitochondrial permeability transition pore (mPTP) (Figure 1) [127]. Mitochondrial Ca^2+^ uptake and release mechanisms are finely equilibrated under resting conditions in order to maintain the matrix Ca^2+^ concentrations at levels similar to cytoplasmic ones. Mitochondria can rapidly take up Ca^2+^ only when microdomains of high Ca^2+^ concentrations occur close to their surface. These Ca^2+^ microdomains are typically formed near the mitochondrial Ca^2+^ channels and receptors. Furthermore, the mitochondria in close proximity to the Ca^2+^ channels of the plasma membrane or the ER are able to rapidly take up Ca^2+^ during cytosolic Ca^2+^ rises [128]. The sites of proximity between the ER and mitochondria constitute specific subcellular regions, called mitochondria-associated ER membrane (MAM) [129]. MAM is a subdomain of the ER that provides a contact site between mitochondria and the ER (Figure 1), and is especially rich in cholesterol and sphingomyelin, thus mimicking the features of lipid rafts [130]. It is involved in various cellular functions, including Ca^2+^ transport, the synthesis of phospholipids, mitochondrial fission and fusion, the division of mtDNA, and cholesterol esterification [131]. The increase in intra-mitochondrial [Ca^2+^] is slow and small for mitochondria that are distant from the microdomains. The alteration of MAMs has been linked to pathological conditions, such as cancer, neurodegenerative diseases, and metabolic syndromes [132].

## 3. Calcium Dyshomeostasis in Alzheimer’s Disease

**Figure 2:**
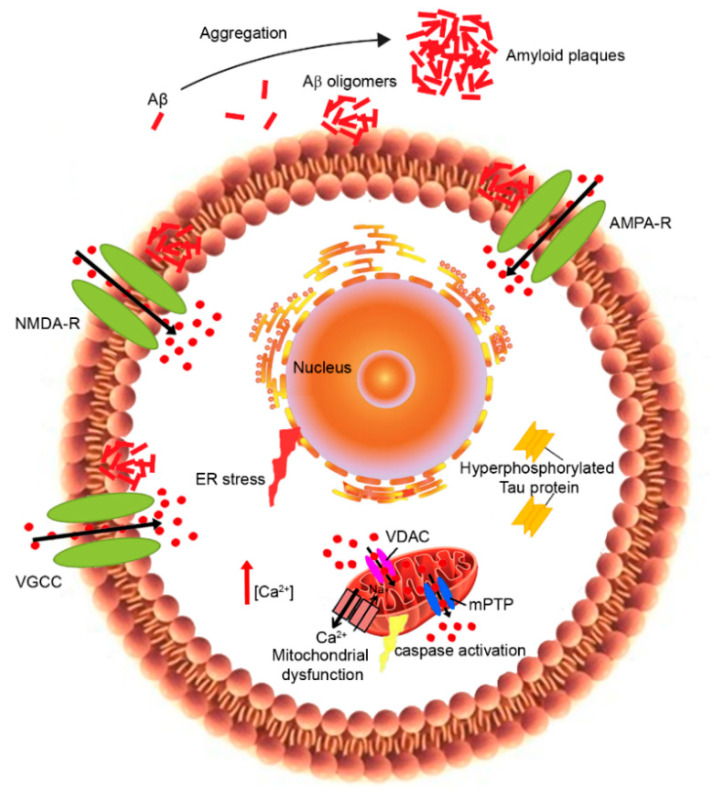
Effects of Aβ and hyperphosphorylated tau protein on Ca^2+^ dysregulation and neuronal dysfunction in AD pathogenesis. Aβ oligomers formed in the extracellular space are able to interact with the plasma membrane, causing the hyperactivation of the calcium channels (NMDAR, AMPAR and VGCC). On the other hand, the intracellular hyperphosphorylated tau protein may promote Ca^2+^ dyshomeostasis. Overall, the increase in cytosolic Ca^2+^ levels results in mitochondrial dysfunction and the subsequent activation of the apoptotic cell death and ER stress.

The so-called “Calcium hypothesis” was first postulated by Khachaturian in 1989 [133] following important experimental studies by the group of [134,135]. It explored how the activation of the amyloidogenic pathway may remodel the neuronal Ca^2+^ signaling pathways responsible for cognition. According to this hypothesis, the depolarization of aged neurons causes the influx of Ca^2+^ from the extracellular space and excitotoxicity. Other studies instead demonstrated that neuronal aging is associated with the alteration of neuronal Ca^2+^ extrusion, leading to old neurons being more vulnerable [136,137,138]. In particular, Ca^2+^ dyshomeostasis has been reported in both peripheral and central neurons during the aging process [136,138] as well as in neurons of AD patients [139,140], influencing both Aβ production and Tau hyperphosphorylation [141,142] (Figure 2). 

Indeed, several studies performed on both AD brains and experimental models showed that the alteration of Ca^2+^ homeostasis occurs before the development of the symptoms, suggesting that Ca^2+^ dysregulation is an upstream event in AD pathogenesis [143]. In addition, the calcium overload was coupled to the deposition of senile plaques and was most pronounced in the immediate vicinity of senile plaques in transgenic mouse models [144,145]. In particular, increased cytosolic Ca^2+^ levels can promote Aβ production and its following neurotoxicity, while the accumulation of the Aβ peptide results in the stimulation of neuronal Ca^2+^ signaling [101]. Therefore, a synergic mechanism between Ca^2+^ and Aβ could intensify the neurodegeneration and cognitive deficits in AD patients [146].

### 3.1. Plasma Membrane Calcium Dysregulation

**Figure 3:**
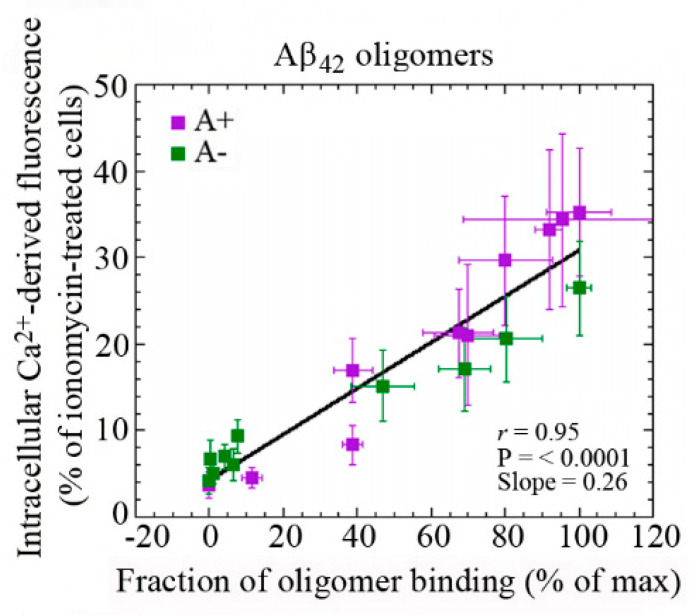
Dependence of intracellular Ca^2+^ dyshomeostasis on the binding affinities of Aβ42 oligomers to cellular membranes with different GM1 content. Changes in the intracellular Ca^2+^ levels plotted against the fraction of oligomer binding to the plasma membrane in GM1-modulated SH-SY5Y neuroblastoma cells treated for 1 h with 10 μM A+ (violet) or A− (green) oligomers of Aβ42 formed according to Ladiwala’s protocol [151]. Reprinted from Figure 3B, Evangelisti et al., 2016, licensed under Creative Commons Attribution 4.0 International Public License (CC BY 4.0; https://creativecommons.org/licenses/by/4.0/ (accessed on 5 May 2021)) [77].

The ability of aberrant protein oligomers to penetrate and disrupt the cellular membrane and induce toxicity appears to result from direct interactions with the lipid bilayers [75,76,147,148,149]. Trodusquemine, a natural product in the aminosterol class, was recently shown to enhance the rate of Aβ aggregation, thus reducing the lifetime or number of toxic oligomeric species. In addition, trodusquemine functions at phisiological concentration to prevent Aβ toxicity by displacyng the aggregates from the cell membranes [148,149]. These studies provide confidence that aminosterols could be useful in the treatment of AD. Our previous analysis has shown the existence of a linear correlation between the rate of Ca^2+^ influx across plasma membranes and the amount of oligomeric species bound to the neuronal surface (Figure 3) [77]. These findings indicate that the susceptibility of neuronal cells to different types of misfolded oligomers is directly related to the extent of the binding of such oligomers to the cellular membrane. The ability of cell membranes to bind oligomeric aggregates appears to depend in turn on the physicochemical properties of both the oligomers and the membranes, which for the latter are determined in large part by their lipid composition [147,150,151]. In particular, the monosialotetrahexosylganglioside GM1 has been found to be an important factor in the context of AD [77,152,153]. GM1, together with cholesterol and sphingomyelin, is abundant in lipid raft domains within the cell membrane that contain a vast array of membrane proteins, including channels and receptors [154].

There is strong evidence of a key role for PrPc, a protein that is associated with lipid rafts, as a receptor for oligomers of the Aβ peptide, resulting in the activation of a Fyn-mediated complex signaling cascade leading to tau phosphorylation and Ca^2+^ dyshomeostasis [155]. Several studies support the idea that oligomers can interact with membranes through direct binding to GM1 [156,157]. This then results in the disruption of lipid bilayers, the alteration of their permeability and the malfunction of raft-associated Ca^2+^ channels, leading to Ca^2+^ influx into cells.

Although there is a variety of experiential evidence, the amyloid channel hypothesis still remains controversial. Indeed, numerous mechanisms can be responsible for the Aβ interaction with neuronal membranes causing the disruption of Ca^2+^ homeostasis. These include the activation of some type of cell surface receptor coupled to Ca^2+^ influx, and the alteration of membrane permeability [147,158]. Several reports showed that alterations in Ca^2+^ levels cause the dysfunction of VGCCs [138], the downregulation of Ca^2+^ clearance mechanisms at the plasma membrane level [136,138] and the failure of Ca^2+^ homeostatic machinery located on intracellular organelles [137,138]. These events affected the maintenance of Ca^2+^ signals in old neurons, impairing learning and memory. It has been shown that Aβ can stimulate the opening of VGCCs, which, in turn, increases the intracellular concentration of Ca^2+^ [159]. In addition, the increase in intracellular Ca^2+^ levels can stimulate the overexpression of Ltype calcium channel subtype (Cav 1.2) in the hippocampal cell membranes of AD models, causing the influx of Ca^2+^ [160]. The inhibition of SERCA as well as the release of Ca^2+^ via the RyR caused the increase in cytoplasmic Ca^2+^. This overloading caused the activation of β-secretase and thus an increased Aβ production and aggregation [115].

In the past few years, in vitro studies demonstrated that the Aβ peptide formed cation-selective pores into the plasma membrane, thus causing Ca^2+^ influx from the extracellular space across these Aβ pore-channels [161,162,163,164]. However, other in vivo studies showed that Aβ can improve the plasma membrane permeability to both anions and cations by altering its dielectric structure [165]. Following this evidence, numerous researchers have focused their attention on the effects of Aβ peptides on Ca^2+^ channels in neuronal cells. In particular, blocking Ca^2+^ influx was found to reduce the neurotoxicity of Aβ oligomers and the levels of insoluble Aβ1–40 and Aβ1–42 in the hippocampus of AD transgenic mice [166]. The increase in intracellular Ca^2+^ promotes the activation of CaN and that of the phosphatases, including PP1, which is involved in long-term depression (LTD) [167]. CaN can contribute together with Aβ or tau to the loss of dendritic spines and synapses, leading to cognitive deficit in AD mouse models. Accordingly, CaN inhibitors can reverse or improve these impairments [168,169]. The Ca^2+^/CaM complex activates the CaMKII, playing an important role in memory formation and synaptic plasticity. Taking into account that many kinases can be activated by Ca^2+^, the dyshomeostasis of Ca^2+^ can increase tau phosphorylation [170,171,172]. Conversely, abnormal accumulation of intracellular tau can also induce Ca^2+^ overload, causing the dephosphorylation of CaMKIV and CREB by activating CaN [168]. The increase in cytosolic Ca^2+^ can also activate JAK2-STAT1 signaling, leading to the binding of STAT1 to NMDARs, and thus inhibits the transcription of specific GAS elements [170]; the increase in the cleaved STAT1 induced by tau also activates BACE1, promoting Aβ production [173]. All of these alterations reveal new mechanisms by which tau can induce synapse impairments and cognitive deficits. PS1 and PS2 have also been implicated in the influx of Ca^2+^, as well as in the ER and mitochondrial Ca^2+^ signaling. Indeed, mutations in these proteins affect Ca^2+^ homeostasis [174].

An altered expression of calcium-binding (CBP) and calcium sensing proteins can modify the calcium-buffering capacity, causing the oversensitivity of neurons to glutamate released in the extracellular space [175,176,177]. In addition, the impairment of glutamate transporter function in the glial cell leads to the accumulation of glutamate in the synaptic cleft, activating the postsynaptic AMPA/NMDA receptors. Thus, Ca^2+^ entry in postsynaptic neurons disrupts the intracellular homeostasis and induces an increase in ROS production. Aβ has been reported to bind to NMDA and AMPA glutamate receptors [178], as well as nicotinic acetylcholine receptors [179], and all of these receptors are highly Ca^2+^-permeable. Furthermore, Aβ can influence VGCCs and IP_3_R [144]. Numerous research studies demonstrated that Aβ promotes the upregulation of VGCCs in different neuronal types, including cortical and hippocampal neurons [180]. In particular, Aβ peptides may over-activate the function of L-type VGCCs through a mechanism involving ROS production [181], or induce the overexpression of CaV1.2 and CaV1.3 channels in the hippocampus of AD transgenic mice [182] and in rat hippocampal neurons treated with Aβ [183]. The effects of Aβ on NMDARs have attracted considerable interest as these ligand-gated channels are involved in synaptic plasticity and LTP [184]. Indeed, Aβ oligomers can induce the overactivation of NMDARs, resulting in a cytosolic Ca^2+^ increase [77,137,153,185,186] through several mechanisms: by affecting glutamate availability [187,188] and/or by modifying NMDAR electrophysiological properties [189], or by changing membrane tension [190]. Importantly, the overactivation of GluN2B NMDAR subunits induced by Aβ [191] has been correlated to ER stress, to the depolarization and dysfunction of mitochondria [192,193], to microtubule disassembly and to a reduction in neurite length [191]. The synaptic activation of NMDAR is crucial for memory formation [194]. Hippocampal neurons showed differential expression of NMDA receptor subunits (NR1, NR2B) in AD-like rats [195]. In particular, the NR2B subunit, which is highly selective for Ca^2+^ transport and is known to play a decisive role in Ca^2+^-induced apoptosis, was overexpressed in AD models compared to controls [195]. The persistent overactivation of the NMDA receptor in the postsynaptic terminal stimulates hippocampal neurons, which allows higher calcium influx resulting in excitotoxicity. The Aβ-induced activation of NMDAR promoted its endocytosis [196]. We have recently observed that lysophosphatidylcholine and arachidonic acid, which cause membrane compression and stretch, respectively, can activate NMDAR and AMPAR through a change in membrane tension induced by Aβ oligomers [190]. In particular, lysophosphatidylcholine is able to neutralize the oligomer-induced activation of the NMDA receptors, whereas arachidonic acid activates the receptors similarly to the oligomers with no additive effects, suggesting that Aβ-induced toxicity can also be caused by the perturbation of the mechanical properties of lipid membranes sensed by NMDA and AMPA receptors [190]. Memantine, an NMDAR antagonist, was approved in 2002 as a therapeutic drug in moderate to severe AD [197,198]. However, other potential drugs targeting NMDARs are not included in AD therapy because of their intolerable side effects.

### 3.2. Endoplasmic-Reticulum Calcium Dysregulation

As reported above, Aβ peptides are able to induce a high release of Ca^2+^ from the ER, thus promoting the activation of the unfolded protein response (UPR) and ER stress [199,200]. Notably, Aβ oligomers may disrupt ER Ca^2+^ homeostasis indirectly by the increase in Ca^2+^ influx from the extracellular space, which in turn triggers Ca^2+^ release from intracellular stores [201], or directly by interacting with several regulators of ER Ca^2+^, such as RyR and I_3_PR [202,203,204,205,206,207]. On the other hand, ER Ca^2+^ dyshomeostasis causes abnormal Aβ production and neuronal apoptosis [208,209,210]. In addition, IP_3_R was found to modulate Ca^2+^ homeostasis in AD [105] and its alterations have been detected in cells derived from AD patients since 1994 [211,212].

### 3.3. Mitochondrial Calcium Dyshomeostasis

Mitochondria play a key role in the modulation of intracellular Ca^2+^ signaling [213]. Indeed, they can quickly resume Ca^2+^ to prevent Ca^2+^ overload into the cytosol, activating the Ca^2+^-dependent mitochondrial matrix dehydrogenase to produce ATP. However, this disruption of mitochondrial Ca^2+^ regulation affects energy production and oxidative stress, resulting in mitochondrial dysfunction [214]. In particular, the excessive Ca^2+^ influx into the mitochondria induces mitochondrial outer membrane permeabilization and the subsequent release of pro-apoptotic factors into the cytoplasm, including cytochrome C and apoptosis-inducing factor, which activate apoptosis cell death [215] (Figure 2). Mitochondrial dysfunction has been proposed as an early event in AD and other aging-related neurodegenerative disorders [216]. Studies on brains from AD patients and AD mouse models showed impaired mitochondrial function, associated with decreased bioenergetics and ATP synthesis [217], morphological abnormalities [218], the imbalance of mitochondrial dynamics [219] and the redistribution of mitochondria [220]. Synapses are particularly rich in mitochondria, which provide energy for Ca^2+^ homeostasis. In addition, synaptic mitochondria are more sensitive to Ca^2+^ dyshomeostasis with respect to non-synaptic mitochondria [221]. During LTP, microtubule associated protein 1B (MAP1B) phosphorylation and local concentrations of CaMKII were increased [222]. CaMKII is responsible for phosphorylating MAP2, which enhances synaptic response [223]. The accumulation of tau impairs synapses and memory by activating Ca^2+^-dependent CaN and suppressing nuclear CaMKIV/CREB signaling, thus revealing a new mechanism by which tau can induce synaptic toxicity [168]. In vitro studies have reported that Aβ oligomers induce mitochondrial Ca^2+^ uptake [224,225] and Ca^2+^ transfer from the ER to mitochondria [226] in cultured rat primary neurons, even if the in vivo mechanism remains unknown. A recent study showed that the increase in Ca^2+^ levels in neuronal mitochondria of transgenic mice appeared only after plaque deposition and before neural death [227]. This mitochondrial Ca^2+^ overload involves toxic extracellular Aβ oligomers and requires the mitochondrial Ca^2+^ uniporter. The authors propose a novel potential therapeutic target for AD through the blocking of the mitochondrial Ca^2+^ uniporter that was found to reduce mitochondrial Ca^2+^ overload [227].

Various studies have observed that the increase in Ca^2+^ levels in the ER caused the leakage of Ca^2+^ into the cytoplasmic compartment, affecting the mitochondrial calcium homeostasis [140,228]. Recently, an interesting hypothesis has been proposed about the role of MAM, the lipid raft-like domain of the ER closely opposed to mitochondria (Figure 1), in AD pathogenesis [229]. As already mentioned, MAM is involved in several important mechanisms, including calcium transport, the synthesis of phospholipids, mitochondrial fission and fusion, the division of mtDNA, and cholesterol esterification [131]. Thus, several studies have been investigating the role of mitochondrial dysfunction mediated by Aβ and MAM, underlying its putative role in AD.

## 4. Concluding Remarks

Many studies have revealed that the perturbation of Ca^2+^ homeostasis is an early event in the cascade of neuronal alterations underlying the cytotoxicity induced by misfolded Aβ aggregates and hyperphosphorylated tau. However, so far, no common consensus has been reached on the molecular mechanisms of neuronal Ca^2+^ overload, causing the remodeling of signaling pathways with excitotoxicity and memory dysfunction in AD. The effects of Aβ on NMDARs have attracted considerable interest as these ligand-gated channels are involved in synaptic plasticity and LTP. Importantly, the over-activation of GluN2B NMDAR subunits induced by Aβ has been correlated to ER stress and to the depolarization and dysfunction of mitochondria. Recently, ER and mitochondrial Ca^2+^ dyshomeostasis have also been proposed as early causative events in AD.

The main unsolved issue is whether the neuronal Ca^2+^ alterations caused by Aβ extracellular deposits could be non-specific, involving just lipid membrane components, or specific by membrane receptors and other cell surface proteins. There is strong evidence of a key role for PrPc, associated with lipid rafts, as a receptor for Aβ oligomers, resulting in the activation of a Fyn-mediated complex signaling cascade, leading to tau phosphorylation and loss of Ca^2+^ homeostasis. However, the data reported in several other studies support the idea that Aβ oligomers can interact with membranes through direct binding to GM1. This then results in the disruption of lipid bilayers, the alteration of their permeability and the misfunction of raft-associated Ca^2+^ channels, leading to Ca^2+^ influx into cells. These findings do not necessarily contradict the view that PrPc behaves as a receptor of a class of Aβ oligomers. Considering the existence of many structurally distinct conformers, different Aβ aggregates could interact with PrPc and GM1 with different affinities. It is increasingly evident that toxicity is not a feature that is inherent to a given type of misfolded protein oligomer, but is instead a property that emerges from the complex interplay between the structural features of oligomers and the lipid composition of the neuronal membranes. Trodusquemine, a natural product in the aminosterol class, was recently shown to prevent Aβ toxicity by displacyng the aggregates from the cell membranes, suggesting that molecules that interact directly with cell membranes, rather than binding oligomeric aggregate themselves, could represent a useful approach in the treatment of AD.

Overall, the question of the molecular basis of Ca^2+^ perturbation in AD pathophysiology needs further investigations for the development of targeted therapies for AD. Memantine, an NMDAR antagonist approved in 2002 as a therapeutic drug in moderate to severe AD, appears to be promising in AD therapy, together with other potential drugs targeting NMDARs and PrPc. However, according to the latest evidence that both Aβ and tau pathologies have synergistic effects, the most efficacious approach to slow AD may be to combine anti-Aβ and anti-tau therapies.
